# Phosphorylation of the PA subunit of influenza polymerase at Y393 prevents binding of the 5′-termini of RNA and polymerase function

**DOI:** 10.1038/s41598-023-34285-7

**Published:** 2023-04-29

**Authors:** Lu Liu, Ramakanth Madhugiri, Vera Vivian Saul, Susanne Bacher, Michael Kracht, Stephan Pleschka, M. Lienhard Schmitz

**Affiliations:** 1 Institute of Biochemistry, Justus Liebig University Giessen, Member of the German Center for Lung Research (DZL), Giessen, Germany; 2 Institute of Medical Virology, Justus Liebig University Giessen, Giessen, Germany; 3 Rudolf-Buchheim-Institute of Pharmacology, Justus Liebig University, Member of the German Center for Lung Research (DZL), Giessen, Germany; 4 German Center for Infection Research (DZIF), Partner Site Giessen, Giessen, Germany

**Keywords:** Infection, Influenza virus

## Abstract

The influenza A virus (IAV) polymerase is a multifunctional machine that can adopt alternative configurations to perform transcription and replication of the viral RNA genome in a temporally ordered manner. Although the structure of polymerase is well understood, our knowledge of its regulation by phosphorylation is still incomplete. The heterotrimeric polymerase can be regulated by posttranslational modifications, but the endogenously occurring phosphorylations at the PA and PB2 subunits of the IAV polymerase have not been studied. Mutation of phosphosites in PB2 and PA subunits revealed that PA mutants resembling constitutive phosphorylation have a partial (S395) or complete (Y393) defect in the ability to synthesize mRNA and cRNA. As PA phosphorylation at Y393 prevents binding of the 5′ promoter of the genomic RNA, recombinant viruses harboring such a mutation could not be rescued. These data show the functional relevance of PA phosphorylations to control the activity of viral polymerase during the influenza infectious cycle.

## Introduction

Influenza viruses are enveloped negative-sense single-stranded RNA viruses with segmented genomes and belong to the family of *Orthomyxoviridae*. Variants of circulating or newly emerging IAVs continue to annually trigger global health threats for both humans and animals and occasionally pandemic outbreaks^1^. IAVs are the most pathogenic subtypes for humans, causing seasonal epidemics and occasionally pandemics^2^. Due to their high genomic plasticity and continuous evolution, they are able to escape host immunity and adapt to new host species^1,3^. IAVs possess an RNA genome of 8 segments that encodes up to 17 proteins^4^. IAVs enter cells by endocytosis and after uncoating the viral ribonucleoprotein complex (vRNP) is trafficked to the nucleus^5^. The vRNP is composed of the virion RNA (vRNA) in association with the nucleocapsid protein (NP) and a heterotrimeric RNA-dependent RNA polymerase (RdRp) complex (composed of PB1, PB2 and PA)^6^. In a pioneering round of transcription, the viral polymerase interacts with host cell RNA polymerase II to allow cap binding of nascent cellular mRNA by PB2 and PA-mediated mRNA cleavage close to the capped 5′ end. The capped RNA cleavage product is then used as a primer for polymerase-mediated generation of viral mRNAs encoding viral proteins^7^. After the production of mRNAs and viral proteins at the beginning of the infection cycle, the polymerase preferentially uses primer-independent replication at later time points, to generate complement copies of vRNA (cRNA) that is stabilized by the binding of freshly synthesized NP protein^8^. The cRNA-containing RNP complexes (cRNPs) then serve as templates for the synthesis of progeny vRNA, which is mediated by newly synthesized viral polymerase dimerizing with cRNP-bound polymerases^9,10^. Both ends of the vRNAs or cRNAs remain partially paired and act as promoters during RNA synthesis^11,12^. The 5′ end of the RNA folds into a stem loop structure that is bound in a pocket formed by PB1 and PA^13–15^.

The temporally offset association of influenza polymerase with vRNA or alternatively with cRNA requires a high degree of regulation. This plasticity is achieved by several mechanisms, which include the adoption of alternative configurations depending on the association with vRNA or cRNA^16^. Subunits of the polymerase complex also interact with numerous host cell proteins where for example association of the polymerase with the S5-phosphorylated C-terminal domain (CTD) of eukaryotic RNA polymerase II is required for generation of viral mRNAs^17,18^. Further examples for regulatory proteins affecting polymerase function are regulatory interactions with p21, sphingosine kinase 1, aryl hydrocarbon receptor nuclear translocator and Ran-Binding Protein 5^19–22^. In addition, the three subunits of the viral polymerase are modified by numerous posttranslational modifications including acetylation, SUMOylation, ubiquitination, Neddylation and phosphorylation^23–29^. The temporally and spatially restricted occurrence of protein modifications such as phosphorylation allow the dynamic and reversible control of many properties that include enzymatic activities and intermolecular interactions.

Here we set out to investigate the functional consequences of phosphorylations occurring at the PB2 and PA subunits of IAV polymerase, which have not yet been investigated. Amino acid changes, which mimic constitutive phosphorylation at PA S395 resulted in a partial limitation of the ability to synthesize mRNA and cRNA. PA phosphorylation at Y393 prevented binding of the 5′-ends of vRNA and cRNA and largely inactivated the polymerase function. These data show the functional relevance of regulated PA phosphorylations during the influenza virus infectious cycle.

## Results

**Figure 1:**
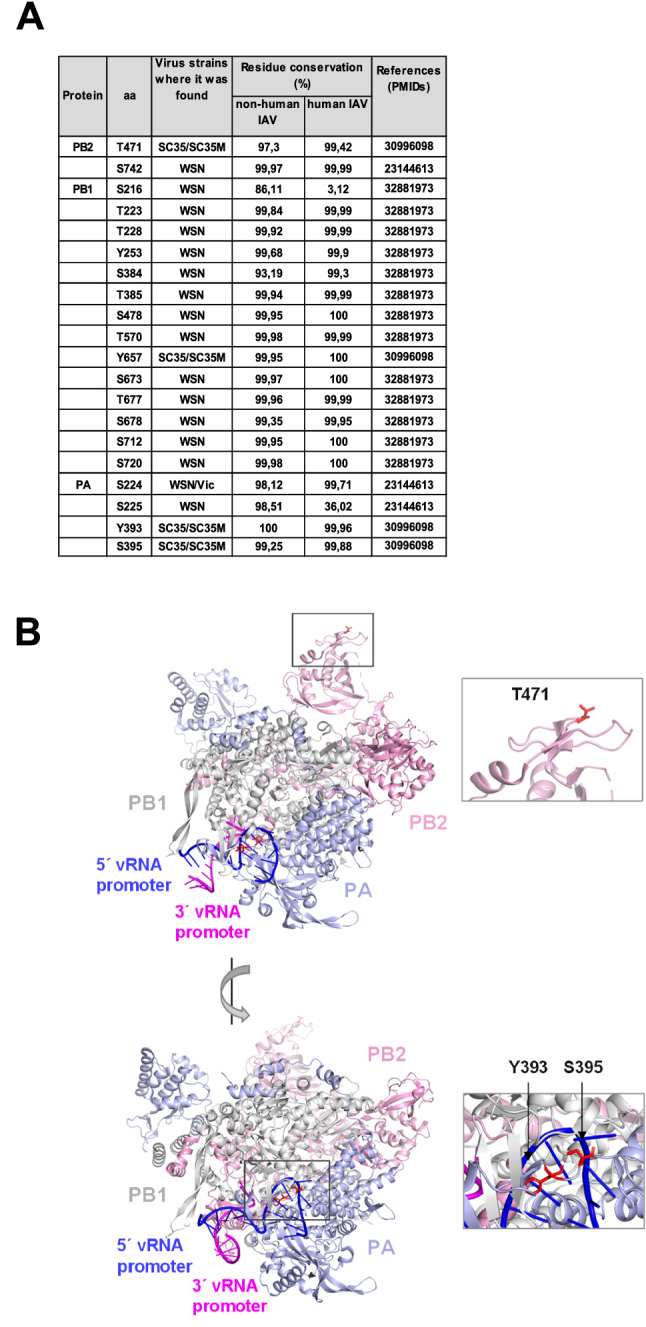
Summary of IAV phosphorylation sites in the polymerase subunits. (**A**) Phosphorylation sites of polymerase subunits occurring in vivo are listed, whereas phosphorylations detected only by in vitro experiments were not included. The virus strains in which the modifications were detected are indicated (WSN: A/WSN/1933 (H1N1); Vic: A/Victoria/3/75 (H3N2); SC35: A/Seal/Massachusetts/1/80 (H7N7); SC35M: A/Seal/Massachusetts/1/80 (H7N7) mouse-adapted). (**B**) The position of PB2 T471 and PA Y393, S395 phosphorylation sites within the polymerase complex are shown in red, the PB2, PB1, and PA subunits and the 5′ vRNA and 3′ vRNA promoters are displayed. The structure is shown in two different degrees of inclination to allow optimal visibility of the phosphorylation sites, boxed areas are enlarged. The structures (PDB: 4WSB) were displayed using Pymol.

**Figure 2:**
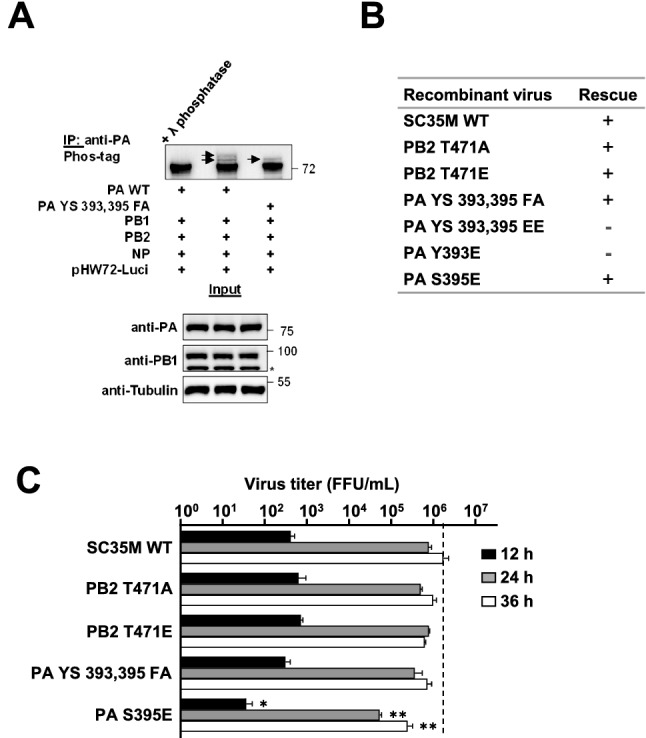
The effects of PB2 and PA phosphorylation on viral replication. (**A**) 293T cells were transfected with pCAGGS vectors directing the synthesis of the four proteins forming the vRNP together with the plasmid pHW72-Luci expressing a Pol1 promoter-driven vRNA-like transcript. One fraction of the extracts was analyzed by SDS-PAGE and immunoblotting for correct expression of the transfected proteins (Input). The remaining material was used for immunoprecipitation and treatment with λ phosphatase as a control, followed by Phos-tag™ SDS-PAGE and detection of PA phosphorylation, as revealed by occurrence of upshifted bands which are indicated by arrows. The position and size (kDa) of molecular weight markers are shown. (**B**) Summary of the rescue of recombinant viruses encoding the indicated WT and mutant proteins, a + indicates successful rescue. (**C**) MLE-15 cells were infected with the indicated viruses at a MOI of 0.001 and viral growth was monitored 12, 24, and 36 h.p.i. by focus forming assays. Focus forming units (FFU) are shown, the dashed line represents the peak of WT SC35M titers. Error bars represent SDs from three independent experiments. All *P* values show significance in comparison to the WT control at each time point using one-way ANOVA (*P ≤ 0.05 and **P ≤ 0.01).

Dynamic regulation of IAV polymerase is also mediated by regulatory phosphorylations that were characterized for the PB1 catalytic subunit^30^. We set out to investigate the possible function of evolutionarily conserved phosphorylation sites located in regions of functional relevance. Interestingly, most of the documented phosphorylation sites of the polymerase subunits are highly conserved between human and non-human IAVs (Fig. 1A). Some of these phosphorylation sites are located in relevant domains including PB2 T471, which is located within the Cap-binding domain (amino acids 319–481)^31^. Two further interesting sites are PA Y393 and S395, which are contained in the so-called PA arch domain, a region contributing to the interaction with PB1 and close to the 5′ vRNA/cRNA binding pocket^13–15^. The positions of these phospho-sites within the IAV polymerase complex are displayed in Fig. 1B. These phosphorylations have been discovered by phosphoproteomic analysis of cells infected with SC35 (derived from A/Seal/Massachussetts/1/80 (H7N7)) and also its mouse-adapted variant SC35M^32^. To confirm the occurrence of these PA phosphorylations by an independent experimental approach, 293T cells were transfected to express all components of a vRNP including the PA wild type (WT) protein or a phosphorylation-deficient mutant (PA YS 393,395 FA). This mutant showed reduced PA phosphorylation, as detected by Phos-tag™ SDS-PAGE (Fig. 2A). To further investigate the potential functional relevance of these phosphorylation sites, we also created phospho-mimetic (S/T/Y to E) mutants. The resulting mutants either lack the occurrence of phosphorylation (phosphorylation-deficient) or the dynamic alternation between the unmodified and the phosphorylated state (phospho-mimetic). These mutated SC35M viruses were produced using a reverse genetics system, as visualized in suppl. Fig. S1. After co-transfection of eight plasmids encoding the viral RNAs and proteins in a co-culture of 293T/MDCK II cells, the resulting virions were amplified on MDCK II cells and the titers were determined. In these experiments, we repeatedly failed in several attempts to generate viruses bearing a phospho-mimetic mutation at PA Y393, either alone or in combination with a further mutation at PA S395 (Fig. 2B, suppl. Table S1). These results suggest that permanent and unregulated phosphorylation at this site prohibits the generation of virus progeny. To characterize the mutations further, all rescued viruses with mutations in the polymerase subunits were investigated for their replication in mouse lung epithelial cells (MLE-15), which are widely used to study SC35M infection^33,34^. A comparative analysis of mutant viruses in multi-cycle replication assays showed roughly similar replication kinetics at 12, 24, and 36 h.p.i. in MLE-15 for all mutant viruses with the exception of the PA S395E mutant, which showed a significantly reduced replication capacity in the early phases of infection (Fig. 2C).

**Figure 3:**
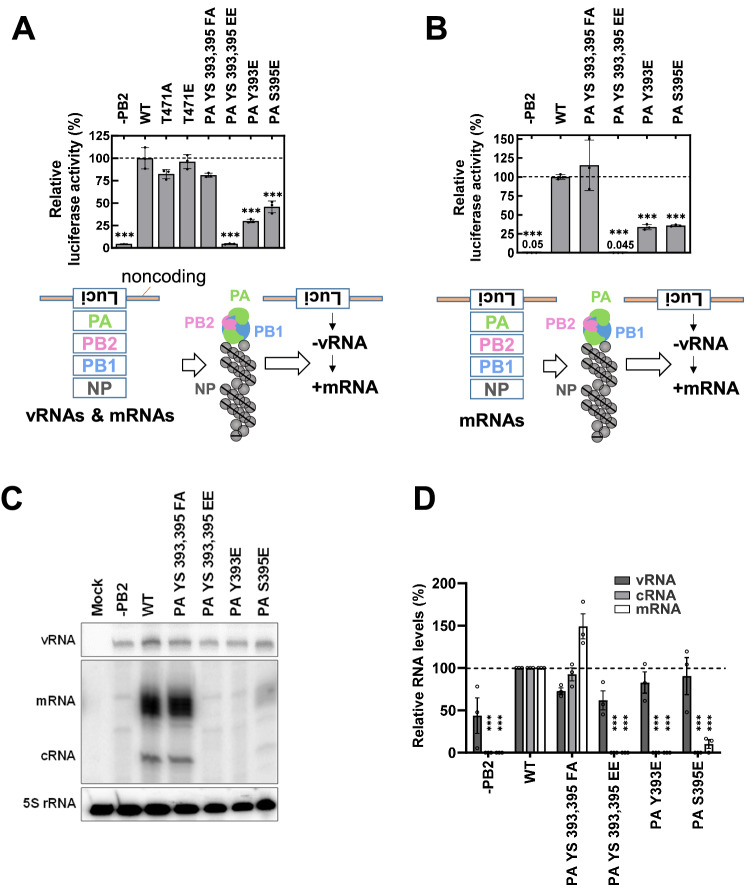
PA phosphorylation impairs RdRp activity. (**A**) The lower part shows the experimental set-up for the minigenome assay co-expressing the v/mRNAs of the viral PB2, PB1, PA and NP proteins. 293T cells were transfected to express the a Pol1-promoter driven vRNA-like transcript encoding the reporter gene together with pHW2000 plasmids allowing the expression of v- and mRNAs encoding the indicated viral proteins. One day later, cells were harvested and activities from Firefly luciferase (generated by vRNP polymerase activity using negative sense vRNA as a template) and Renilla luciferase (generated by a constitutive RNA polymerase II promoter and used for normalization) were determined. Activity by the WT vRNP was set to 100%, error bars represent SDs from three independent experiments. (**B**) The experiment was done as in (A) with the difference that the viral proteins were expressed from pCAGGS plasmids, not generating vRNAs, as schematically shown in the lower part. Extracts were used for determination of luciferase activity, the numbers show relative activity compared to the WT polymerase. (**C**) 293T cells were transfected with pCAGGS vectors directing the synthesis of the four proteins forming the vRNP together with the pPOLI-CAT-RT reporter plasmid, which generates a virus-like RNA as a first template. After isolation of RNA, the levels of cRNA, mRNA, rRNA (loading control) and the input vRNA (derived from a constitutive RNA polymerase I promoter) were determined by extension of ^32^P-unlabeled primers, gel electrophoresis and autoradiography. (**D**) Data from three independent experiments performed in (**C**) were quantified and normalized to the 5S rRNA control. The RNA levels determined in cells expressing the WT vRNP were set to 100%. In all experiments, *P* values ≤ 0.001 are indicated by ***.

To test whether the modifications occurring at the polymerase subunits PB2 and PA affect activity of viral polymerase, minigenome replication assays using a luciferase reporter gene were performed. In this system, a set of bidirectional plasmids co-expressing a vRNA-like transcript and a corresponding mRNA, both encoding the SC35M PB1, PB2, PA or NP proteins were co-expressed with a reporter gene. The reporter plasmid directs the production of a negative-sense vRNA-like transcript, which can be converted from the vRNP to a luciferase encoding mRNA, thus providing a quantitative read-out for RdRp activity, as schematically shown in Fig. 3A. To determine the impact of the selected PA and PB2 phosphorylation sites on RdRp polymerase activity, 293T cells were transfected with the reporter plasmid along with four plasmids encoding NP and the WT polymerase proteins or their phospho-mimetic or phospho-deficient PB2 and PA mutants. Quantification of the luciferase activity did not reveal any significant contribution of PB2 T471 phosphorylation for polymerase function, while the phospho-mimetic mutations of PA S395 led to a significant reduction in RdRp activity. Even less activity was found for the PA Y393E mutant, while mutation of both sites resulted in loss of RdRp polymerase activity (Fig. 3A). As the selected PA phosphorylation sites are relevant for RdRp-mediated transcription, they were further analyzed using an alternative mini-genome reporter system^35^. This experimental setup differs from the aforementioned system in that the viral proteins are expressed only from mRNA without the corresponding vRNA (see Fig. 3B). Also these experiments confirmed the impaired transcriptional activity of the PA Y393E and S395E mutants. Together, these experiments suggest that constitutive phosphorylation at Y393 and/or S395 impairs polymerase-mediated mRNA production, but do not allow any conclusions on the production of cRNA.

To address this question, primer extension experiments were performed, which correspond to the experimental scheme shown in Fig. 3B with the exception that a pPOLI-CAT-RT reporter plasmid was used^35^. This reporter is transcribed by RNA Polymerase I to generate a vRNA-like transcript that can be converted by the co-expressed viral polymerase to yield cRNA and mRNA, respectively^36,37^. Cells were transfected as shown, followed by isolation of RNA and detection of the different RNA species by extension of specific radiolabeled primers. Gel electrophoresis and autoradiography allowed to detect similar levels of RNA polymerase I-derived vRNA as a template as expected, but strong differences in the production of mRNA and cRNA (Fig. 3C). No mRNA and cRNA was produced by polymerases with a PA Y393E mutation, while the PA S395E mutant showed residual generation of mRNA and cRNA. Together, these results and their quantitative evaluation (Fig. 3D) suggest that the inability to rescue recombinant SC35M viruses expressing the PA Y393E or the PA YS 393, 395 EE mutants is attributable to a defective polymerase function.

**Figure 4:**
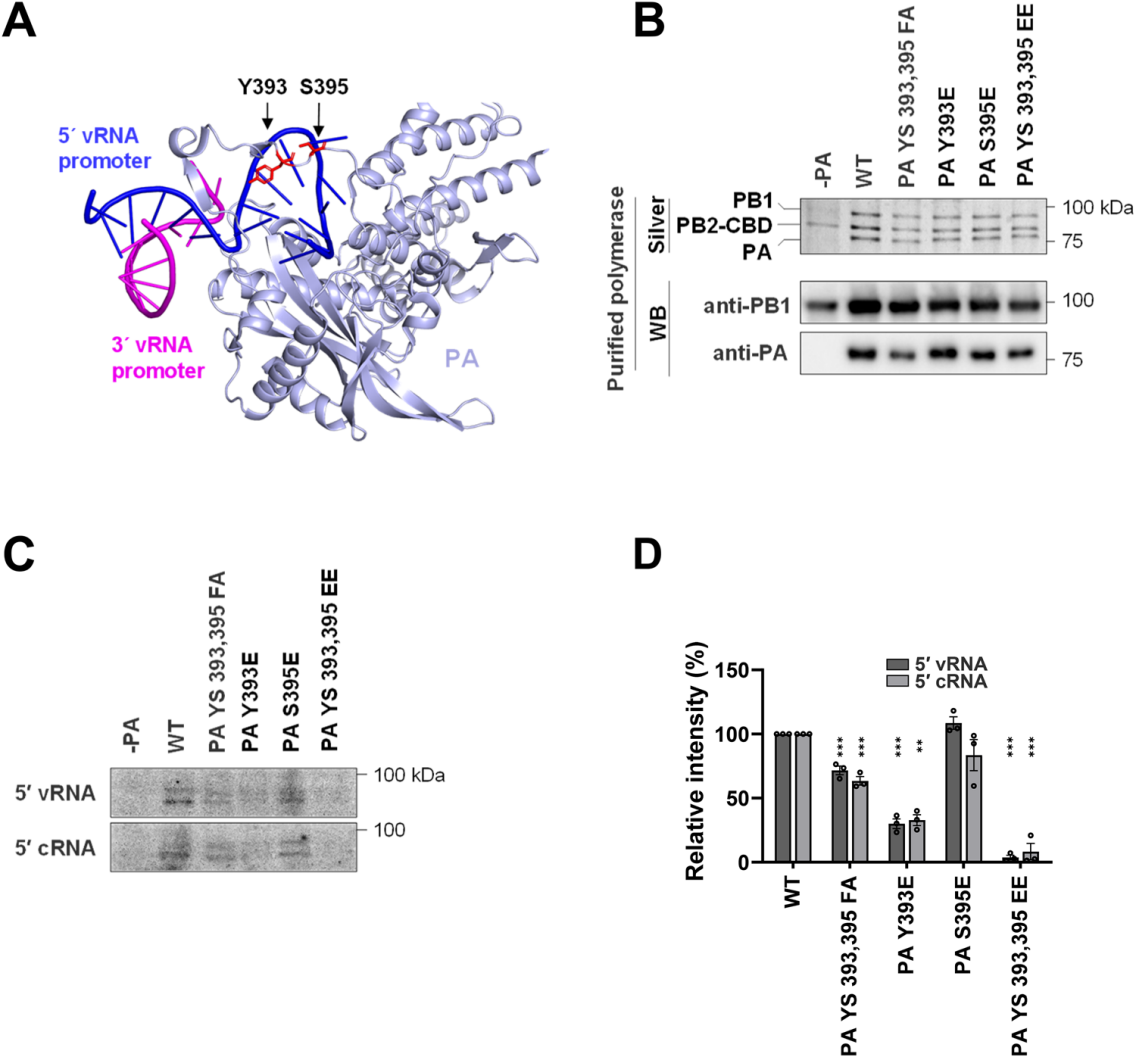
PA Y393 phosphorylation prevents polymerase binding to the 5′-termini of vRNA and cRNA. (**A**) For convenient visibility only the PA subunit and the positions of Y393 and S395 phosphorylation sites are shown, the 5′ end of the vRNA forming a stem-loop is displayed in blue. The structures were displayed using Pymol (PDB: 4WSB). (**B**) The trimeric polymerase complex was expressed in 239T cells and purified using the TAP tag. The purified material was analyzed for purity and integrity of the polymerase subunits by silver staining (upper) and immunoblotting (lower) as shown. (**C**) The purified polymerase was incubated with the radiolabeled 5′ ends of vRNA or cRNA in the presence of their respective unlabeled 3′ promoters. After UV crosslinking the RNA/polymerase complex, the proteins were resolved by SDS-PAGE and the dried gel was analyzed by phosphorimaging. (**D**) Quantitative analysis of three different RNA-binding experiments, each performed with a different preparation of purified IAV polymerase. After subtraction of background obtained in the control lacking the PA protein the data were normalized to the silver-stained input control, the intensity of the WT polymerase was set to 100%. Data are mean ± SEM (**P ≤ 0.01 and ***P ≤ 0.001).

What molecular mechanism can explain the lack of functionality of the Y393-modified PA subunit? Y393 and also the neighboring S395 are part of the PA arch, an extended loop through which a ß-hairpin region of the PB1 protein inserts^14^. To investigate whether the mutant PA Y393E protein shows any changes in its ability to interact with PB1, co-immunoprecipitation experiments were performed. The PA Y393 phosphorylation status had no consistent and statistically significant effect on its ability to interact with PB1, regardless whether both proteins were expressed alone, together with PB2 (suppl. Fig. S2A) or even in the presence of all components of the RNP including vRNA (suppl. Fig. S2B). Similarly, PA phosphorylation had no discernable effects on the intracellular localization of PA (suppl. Fig. S3). The PA sheet and the inserted PB1-ß hairpin together form an integral part of the binding site for the 5′ ends of vRNA and cRNA containing a stem-loop structure^38,39^, as visualized for the PA/RNA interaction in Fig. 4A. To determine the effects of PA phosphorylation on its ability to bind RNA, we measured the interaction between the purified polymerase complex and the 5′ ends of vRNA and cRNA. These in vitro experiments followed established protocols where the polymerase complex is purified by tandem affinity purification (TAP)^40,41^. Fusion of the TAP-Tag to the C-terminus of SC35M PB2 unexpectedly precluded its expression upon co-expression of further members of the polymerase (suppl. Fig. S4) for unknown reasons. We therefore mutated the evolutionary conserved Y393 and S395 sites in the PA protein from the A/WSN/33 (H1N1 WSN) strain and expressed the affinity-tagged PB2 protein along with the WSN PB1 and PA and its mutants in HEK 293T cells. The polymerase complex was isolated by affinity purification and further characterized by silver staining and immunoblotting (Fig. 4B). Equal amounts of purified polymerase complexes were incubated with ^32^P-labeled 5′ vRNA and cRNA promoter in the presence of the corresponding unlabeled 3′ RNA promoters, followed by UV irradiation for covalent linkage of RNA/protein complexes. After SDS-PAGE the amount of 5′-RNA-bound RdRp was measured by phosphoimaging (Fig. 4C) and its quantitative evaluation (Fig. 4D). These experiments showed largely unchanged RNA-binding for PA S395E, while RNA-binding was severely compromised upon phospho-mimetic mutation of Y393 either alone or in combination with S395.

## Discussion

The regulation of RNA polymerase function by phosphorylation is a widely used principle occurring from yeast to humans^42^. For influenza polymerases, evidence for direct phosphorylation-mediated control of RdRp activity was obtained only 2020^30^. This study showed that different phosphorylations occurring on the catalytic PB1 subunit control either RNA binding, transcription or alternatively have no apparent function, similar to the PB2 T471 phosphorylation investigated here. Our data indicate that phosphorylation of PA at Y393 and S395 lead to impaired polymerase function. Minigenome reporter assays and primer extension analyses showed that phosphorylation at S395 leaves remaining polymerase function, and consistently virions harboring the PA S395E mutant could be rescued. The effects of the S395E mutation on transcription were more pronounced in the in vitro system (Fig. 3C) than in the cell-based reporter assays (Fig. 3A, B), suggesting the presence of compensatory mechanisms within the cell that might include changes in protein/protein interactions or conformation. The transcriptional defect caused by the mutation mimicking permanent Y393 phosphorylation is more severe and introduction of phospho-mimetic amino acids at Y393 and S395 completely shuts down the activity of the viral polymerase. Such a double mutation might also affect the overall structure of the PA arch, consistent with the notion that also the PA YS 393, 395 FA mutant shows reduced RNA interaction. Although the interactions between the polymerase subunits are largely unaffected by PA phosphorylations (see suppl. Fig. S2) we cannot rule out potential effects on the different conformation states that the holoenzyme can adopt. Since the modification of tyrosine and serine is mediated by different kinases^43^, individual modification of the respective side chains would be a suitable mechanism to gradually decrease RdRp activity. The analysis of phospho-mutants for PB1^30^ and also PA (this study) showed that phospho-deficient mutants are more tolerated than phospho-mimetic mutants. This is consistent with the notion that regulated activities are mediated by dynamically altered phosphorylations, which is ensured by the balanced activity of kinases and counteracting phosphatases^44^. This study shows that inactivation of polymerase function by PA Y393 phosphorylation will involve impaired binding of vRNA and cRNA, as it was also predicted for PB1 phosphorylation at S673^30^. PA phosphorylation at S395 reduces activity of the viral polymerase without affecting RNA binding, suggesting that this modification might affect other features of the polymerase. This modification might influence the 3D-structure of the polymerase, which can assume discrete conformational states to allow different positioning of the distinct RNA templates^15,45,46^. In principle, PA modification could also affect further features of the polymerase, as it was revealed by the analysis of PB1 phosphorylation. While PB1 phosphorylation at S673 selectively deactivated mRNA production without affecting replication, other modifications either had no discernible effect (S216 and S384) or completely blocked RdRp activity (S478 and T223)^30^. These phosphorylations most likely do not represent an antiviral strategy of the host cell, since the modification sites are evolutionarily conserved although they can be mutated to phospho-deficient residues without loss-of-function. It is therefore possible that modifications of this multifunctional machine control its different activities during viral infection such as the switch from mRNA production to cRNA generation. However, similar to eukaryotic RNA polymerase, also initiation and elongation of transcription could be controlled by phosphorylations. While there are numerous publications on the effects of protein kinases and phosphatases on IAV polymerase function^47–50^, studies on the importance of individual phosphorylations on polymerase function are still in their infancy, raising a number of unresolved questions. As the polymerase is simultaneously modified by multiple phosphorylations at various subunits, it will be relevant to identify these modification patterns occurring for the endogenous polymerase in a time-resolved fashion, thus allowing to investigate their interplay and functional relevance. Furthermore, it would be exciting to learn to what extent polymerases engaged in the production of mRNA, cRNA or vRNA differ not only in their conformation^16^, but possibly also in their phosphorylation patterns.

## Materials and methods

### Antibodies, primers and reagents

This information is given in suppl. Table S2.

### Cell culture

Human embryonic kidney 293T cells (293T, ATCC: CRL-3216), Madin-Darby and canine kidney cells (MDCK II, ATCC: CRL-2936) and murine MLE-15 lung epithelial cells (Cellosaurus: CVCL_D581) were grown in Dulbecco’s modified Eagle medium (DMEM) supplemented with 10% (v/v) fetal calf serum, 100 U/ml penicillin and 100 μg/ml streptomycin. Cells were grown in an incubator at 37 °C in a humidified atmosphere containing 5% (v/v) CO_2_.

### Sequence alignment and structural modeling

All previously published protein sequences of non-human IAV and human IAV PB2, PB1, and PA (before August 2022) were collated from the Influenza Virus Resource at the National Center for Biotechnology Information (NCBI)^51^. Laboratory strains were excluded from the analyses to eliminate sample biases. Identical sequences were collapsed and filtered to ensure that each strain was only represented once in the dataset. All assembled protein sequences were aligned using MAFFT v 7.505 as available in the Cipres Science Gateway (https://www.phylo.org) with subsequent manual adjustment to correct frame shift errors using BioEdit. The downstream analyses were based on a data set of 31,276 of PB2, 31,526 of PB1, 31,523 of PA sequences isolated from non-human species, 33,084 of PB2, 32,050 of PB1, 32,933 of PA human IAV sequences. Protein structures (PDB accession number 4WSB) were analyzed using Pymol (version 2.4.1).

### Generation of recombinant IAVs

Recombinant SC35M viruses were generated by using a set of eight plasmids based on the bidirectional pHW2000 plasmid reverse genetics system^32^. A co-culture of 293T and MDCK II cells (ratio 3:1) was used, as these cells are suitable and frequently used to produce recombinant SC35M viruses^32,34,52^. Cells were grown to 70% confluence and transfected with 1 μg DNA of each plasmid encoding the eight viral segments by using Opti-MEM (Life Technologies) and Lipofectamine 2000 (Invitrogen). The medium was removed 6 h post transfection (h.p.t.), and fresh DMEM supplemented with 0.2% (w/v) BSA, 100 U/ml penicillin and 100 μg/ml streptomycin (Life Technologies) was added. To monitor the successful de novo propagation of IAVs, the transfected dishes were screened for the appearance of a cytopathic effect (CPE) each day. The cell culture supernatant was harvested 48 h.p.t. and cell debris was removed by centrifugation. A 500 μl aliquot of each supernatant was used to infect MDCK II cells, which were then further incubated for 48–72 h. The viruses rescued from these cells were titrated by foci assays and stored at − 80 °C until further use, the successful introduction of mutations was confirmed by sequencing.

### Virus infection and titer determination

For multi-cycle infections, MLE-15 cells were infected with SC35M viruses in PBS++/BSA (PBS containing 0.2% (w/v) BSA, 1 mM MgCl_2_, 0.9 mM CaCl_2_, 100 U/ml penicillin and 100 μg/ml streptomycin (Life Technologies)) at a multiplicity of infection (MOI) of 0.001. After adsorption for 1 h at room temperature (RT), the inoculum was removed and monolayers were washed three times with PBS++. An appropriate amount of infection medium (DMEM supplemented with 0.2% (w/v) BSA and penicillin/streptomycin) was added and cells were incubated for the indicated periods at 37 °C. Infectious particles in the cell supernatants were quantified by focus forming assays on MDCK II cells grown in 96-well plates. These were infected with 20 μl virus samples serially diluted in PBS++ (dilution from 10^–1^ to 10^–7^) for 1 h at RT. The inoculum was replaced by 150 μl Avicel-medium (MEM supplemented with penicillin/streptomycin, 0.3% (w/v) BSA, 0.3% (w/v) NaHCO_3_, 0.01% (w/v) DEAE Dextran, 1.25% (w/v) Avicel^®^). The cells were further incubated for 24 h at 37 °C and 5% (v/v) CO_2_. To detect foci of infected cells resulting from an infectious particle, cells were fixed and permeabilized with 150 μl fixing solution (4% (w/v) paraformaldehyde and 1% (v/v) Triton X-100 (Roth, Germany) in PBS++) for 1 h at RT. The solution was then discarded and cells were washed 3× with PBS++ containing 0.05% (v/v) Tween-20. The cells were then incubated with primary antibody (50 μl/well) recognizing the IAV NP protein diluted 1:100 in PBS++ containing 3% (w/v) BSA for 1 h at RT. Then cells were washed 3× with PBS++ containing 0.05% (v/v) Tween-20 and incubated with 50 μl/well horseradish peroxidase-coupled secondary antibody diluted 1:1000 in PBS++ containing 3% (w/v) BSA, for 1 h at RT. Cells were then washed 3× with PBS++ containing 0.05% (v/v) Tween-20 and incubated with 40 μl staining solution for 40 min at 37 °C in the dark. After staining, the substrate was removed and cells were washed 2× with H_2_O to remove salts. To detect and quantify foci, the 96-well plates were scanned using a BioSys Bioreader^®^ (BioSys, Karben, Germany).

### Cell-based minigenome luciferase assays

Minigenome experiments were performed to investigate the activity of the reconstituted polymerase and its mutated forms in 293T cells. Minigenome assays co-expressing viral v/mRNAs encoding the SC35M PB2, PB1, PA and NP were done upon transfection of 293T cells (6-well plates, 2 × 10^5^ cells/well) with either 1 μg each of pHW2000-SC35M-PB2, pHW2000-SC35M-PB1, pHW2000-SC35M-PA, pHW2000-SC35M-NP, together with 200 ng of a plasmid expressing a Pol1-promoter driven vRNA-like transcript encoding the firefly luciferase reporter gene (pHW72-Luci) and 20 ng pCI-neoRenilla-Luci. Minigenome assays expressing only the viral RdRp and NPproteins were done in a similar way by transfection of pCAGGS plasmids encoding PB2, PB1, PA (each 125 ng) and NP (800 ng) together with 200 ng pHW72-Luci and 20 ng pCI-neoRenilla-Luci. Transfection was performed using linear polyethylenimine for 5 h as described^53^. 24 h.p.t. the cells were harvested, washed, lysed for 15 min in 150 μl of 1× passive lysis buffer. Firefly and Renilla luciferase activities were detected using the Dual-Luciferase^®^ reporter assay system (Promega) according to the manufacturer's protocol. Renilla activities were used for data normalization and expression of the viral proteins was ensured by Western blotting.

### RNA isolation and primer extension analysis

For the in vitro reconstitution of the SC35M polymerase, five plasmids were co-transfected into 293T cells in 6-well plates. The first plasmid, pPOLI-CAT-RT, contains the CAT open reading frame in minus sense as a reporter gene, flanked by the 3′ and 5′ noncoding regions of the NS RNA segment of influenza virus A/WSN/33 (H1N1). Expression of the influenza virus-like RNA is driven by a human RNA Pol I promoter and a ribozyme sequence that generates the desired 3′ end by autocatalytic cleavage. The other four plasmids, pCAGGS-PB1, -PB2, -PA, and -NP express the influenza virus SC35M PB1, PB2, PA, and NP proteins. The total RNA was extracted using Trizol reagent (Invitrogen, USA) at 48 h.p.t. and carried out as previously described^54^. Briefly, RNA was reverse transcribed with SuperScript III reverse transcriptase (Invitrogen) using specific ^32^P-labeled primers detecting the CAT reporter (v-, c-, mRNA) and a primer for cellular 5S rRNA as a loading control. The products were resolved on a 8% polyacrylamide gel containing 7 M urea and visualized by phosphorimaging on a Amersham™ Typhoon™ instrument.

### Co-IPs, SDS-PAGE and Western blotting

pCAGGS plasmids encoding PB2, PB1, PA (each 1.5 μg), NP (3 μg), and a Pol-1 promoter-driven vRNA-like firefly luciferase-encoding transcript pHW72-Luci (500 ng) were expressed in 293T cells at the indicated combinations. Cells were lysed 48 h later in IGEPAL buffer (20 mM Tris/HCl, pH 7.4, 150 mM NaCl, 1% (v/v) IGEPAL CA-630 (Sigma), 10% (v/v) glycerol, 1 mM phenylmethylsulfonylfluoride, 10 mM NaF, 0.5 mM sodium orthovanadate, 1 µg/ml leupeptine, and 2 µg/ml aprotinin) on ice for 20 min. The lysates were cleared by centrifugation for 10 min at 4 °C and 16,000×*g*. The supernatant was then used for IPs, while 10% of the supernatants were collected as input controls. IPs were performed in the presence of 25 µl protein A/G agarose beads (Millipore) and 1 µg of anti-PA antibody. After rotation for 4 h at 4 °C, beads were washed five times with 1 ml IGEPAL buffer at 4 °C for 10 min and eluted in 1× SDS sample buffer. Denaturing SDS-PAGE was performed as described^53^, PA phosphorylation was determined in the presence of Phos-tag™ acrylamide (FUJIFILM Wako) in the separating gel. Following SDS-PAGE, proteins were transferred to a PVDF membrane as described^55^, immunoreactive bands were detected using the Western Lightning Plus-ECL reagent (Perkin Elmer) and visualized on a ChemiDoc Imaging System (Bio-Rad).

### Purification of recombinant IAV polymerase and RNA cross-linking

293T cells were grown on 10 cm dishes and transfected with 3 μg of each of pcDNA-WSN-PB2-TAP, pcDNA-WSN-PB1, and pcDNA-WSN-PA using linear polyethylenimine. After 2 days, cells were washed in PBS and lysed in 500 μl lysis buffer (50 mM Tris–HCl pH 8.0, 200 mM NaCl, 25% (v/v) glycerol, 0.5% (v/v) IGEPAL CA-630, 1 mM dithiothreitol (DTT), 1 mM phenylmethylsulfonylfluoride, 1 µg/ml leupeptine, and 2 µg/ml aprotinin) and placed on a rotating wheel at 4 °C for 30 min. The lysate was centrifuged at 17,000×*g* for 10 min, and the supernatant was diluted with 2 ml 150 mM NaCl together with 50 μl pre-washed IgG Sepharose 6 Fast Flow beads, followed by rotating at 4 °C for 2 h. The beads were then collected and washed three times with cold wash buffer (10 mM Tris–HCl (pH 8.0), 150 mM NaCl, 10% (v/v) glycerol, 0.1% (v/v) IGEPAL CA-630, 1 mM phenylmethylsulfonylfluoride) and once with cold cleavage buffer (10 mM Tris–HCl (pH 8.0), 150 mM NaCl, 10% (v/v) glycerol, 0.1% (v/v) IGEPAL CA-630, 1 mM DTT, 1 mM phenylmethylsulfonylfluoride). The bound IAV polymerase complex on the IgG beads was then resuspended in 200 μl cleavage buffer and released by incubation with 2.5 μl TEV protease (NEB #P8112) on a rotating wheel at 4 °C overnight to remove the protein A domains, but leaving a calmodulin-binding domain (CBD). The beads were then centrifuged at 17,000×*g* for 5 min, and the eluates containing the IAV polymerase complex were collected, aliquoted, flash frozen in liquid nitrogen and then stored at – 80 °C until use. The protein levels of partially purified recombinant IAV polymerase were analyzed by SDS-PAGE and silver staining using a SilverXpress kit (Invitrogen). The radioactive capped 5′ vRNA/cRNA promoter binding to the IAV polymerase was set up in a 10 μl reaction, as described previously^40^. Purified IAV polymerase was incubated for 20 min at 30 °C with 1 mM DTT, 2 U/μl RNasin (Promega) together with 5 × 10^4^ cpm [γ^32^P]-labeled 5′ vRNA promoter (5′-AGUAGAAACAAGGCC-3′) and 0.5 μM of the unlabeled 3′ vRNA promoter (vRNA: 5′-GGCCUGCUUUUGCU-3′. Alternatively the polymerase was incubated with 5 × 10^4^ cpm [γ^32^P]-labeled 5′ cRNA promoter (5′-AGCAAAAGCAGGCC-3′) together with 0.5 μM of the unlabeled 3′ cRNA promoter (5′-GGCCUUGUUUCUACU-3′). The reaction mixtures were then transferred into a 96-well plate and UV irradiated (254 nm) using a UV Stratalinker 1800 (Stratagene) for 10 min. Cross-linked complexes were separated by 8% SDS-PAGE and visualized by phosphorimaging on a Amersham™ Typhoon™ instrument.

### Quantification and statistical analysis

Image Lab 6.0.1 (Bio-Rad) was used for image acquisition and densitometric analysis of Western blot data. Quantification of ^32^P signals acquired by the Amersham™ Typhoon™ platform was done by subtracting the background values "-PB2" control (primer extension) or "-PA" control (RNA-binding). Data were normalized to loading controls and GraphPad Prism 9 (GraphPad Software, La Jolla, CA, USA) was used to perform statistical analysis and generation of graphs. The statistical significance for multiple comparisons was calculated using a one-way ANOVA followed by Dunnett’s post hoc analysis of pair-wise comparisons to WT using Graphpad Prism 9.

### Immunofluorescence staining

293T cells were seeded on poly-l-lysine-coated coverslips and transfected with expression plasmids as indicated in the figure legend. One day post-transfection, cells were washed with PBS and fixed with 4% (v/v) paraformaldehyde at room temperature for 10 min. The cells were rinsed three times in PBS and blocked for 1 h at room temperature with 3% (w/v) BSA in PBS containing 0,3% (v/v) Triton X-100. Coverslips were subsequently incubated with the anti-PA antibody, diluted 1:2000 in PBS containing 0.3% (w/v) BSA and 0.3% (v/v) Triton X-100, for 90 min at room temperature. After washing three times with PBS + 0,3% (v/v) Triton X-100 cells were incubated with the secondary Alexa488-conjugated antibody (Jackson ImmunoResearch, 1:3000 dilution) in the dark. After 1 h incubation, cells were washed three times in PBS and the nuclear DNA was stained with Hoechst 33324 (Invitrogen). Coverslips were mounted with Mowiol mounting medium and stored at 4 °C. Stained cells were analyzed using an Eclipse TE2000-E microscope (Nikon) and a 63× oil-immersion lens. At least > 30 healthy interphase cells of three independent experiments were analyzed and pictures were taken with an OCRA-spark digital CMOS camera (C11440-36U, Hamamatsu).

### Biosafety

All experiments using infectious virus were performed in accordance with German biosafety regulations pertaining to the propagation of IAVs. All experiments involving IAVs were performed using Biosafety Level 3 containment laboratories approved for such use by the local authorities (RP, Giessen, Germany).

## Supplementary Information

Supplementary Figures.

Supplementary Table S1.

Supplementary Table S2.

Supplementary Information 4.

## Data Availability

Requests for datasets or reagents should be directed to the corresponding author (M.L.S.).
