# Supplementary material for: Phosphorylation of the PA subunit of influenza polymerase at Y393 prevents binding of the 5′-termini of RNA and polymerase function
Source: Sci Rep. 2023 Apr 29;13:7042. doi: 10.1038/s41598-023-34285-7 (PMC10148841; doi:10.1038/s41598-023-34285-7)
Supplement: Supplementary file 1 — Supplementary Figures. [file 41598_2023_34285_MOESM1_ESM.pdf]

Phosphorylation of the PA subunit of influenza polymerase at Y393 prevents binding of the 5'-termini of RNA and polymerase function

Lu Liu[1,2], Ramakanth Madhugiri[2], Vera Vivian Saul[1], Susanne Bacher[1], Michael Kracht[3], Stephan Pleschka[2,4] and M. Lienhard SCHMITZ[1,*]

[1]Institute of Biochemistry, Justus Liebig University Giessen (Germany), Member of the German Center for Lung Research (DZL)

[2]Institute of Medical Virology, Justus Liebig University Giessen (Germany)

[3]Rudolf-Buchheim-Institute of Pharmacology, Justus Liebig University, Giessen (Germany), Member of the German Center for Lung Research (DZL)

[4]German Center for Infection Research (DZIF), partner site Giessen (Germany)

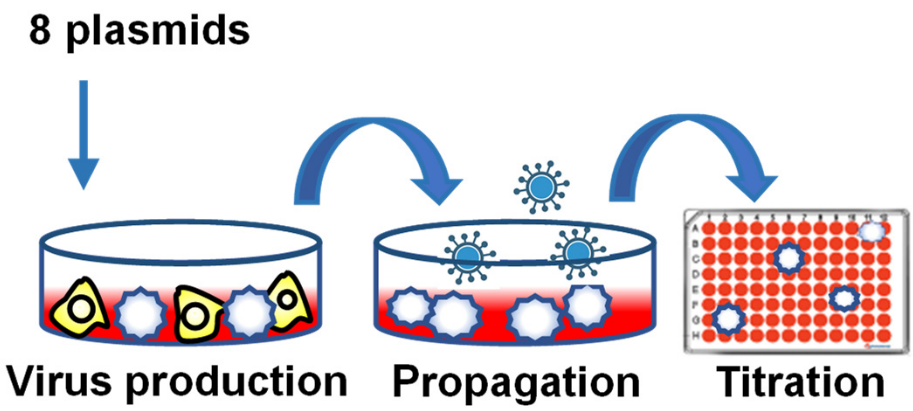

Suppl. Fig. S1. Schematic display of the workflow for the production of recombinant IAV SC35M viruses encoding mutated viral proteins.

A

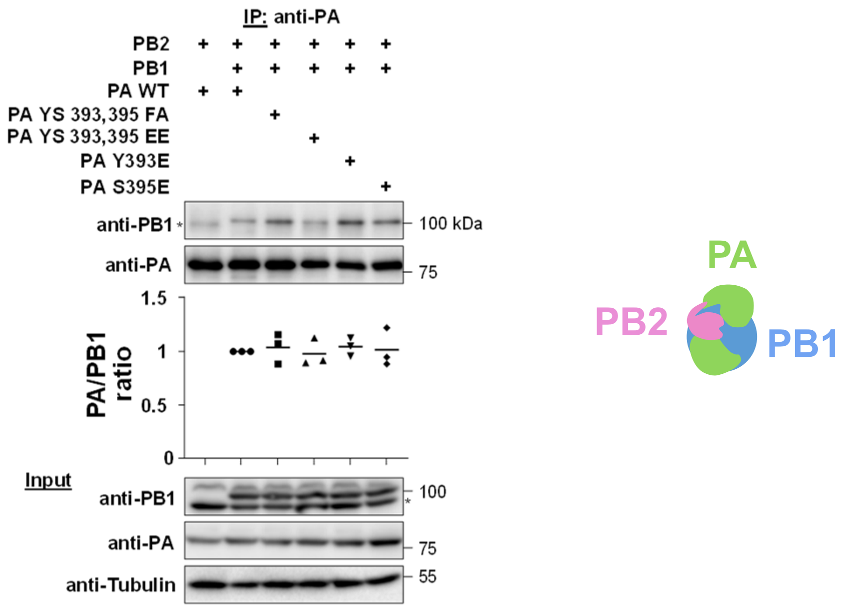

B

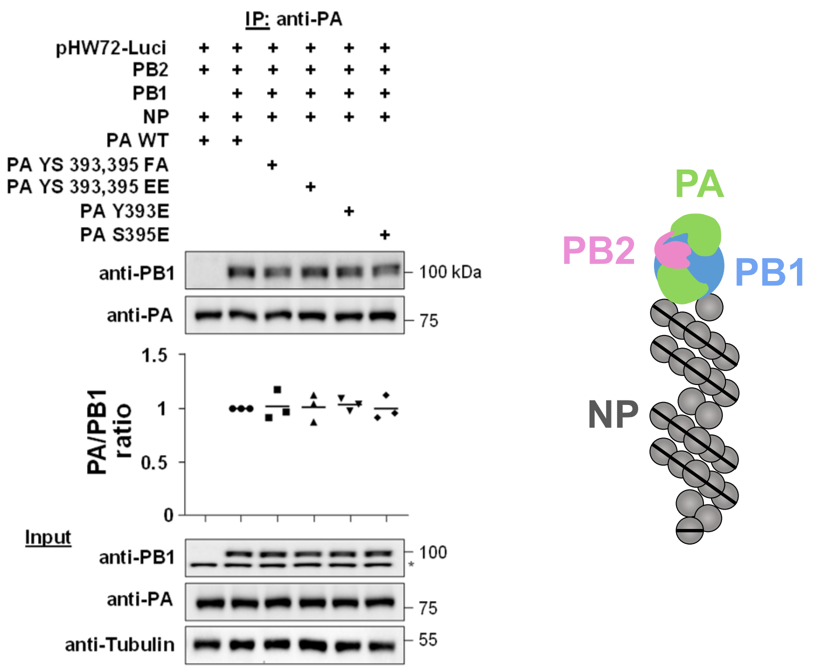

Suppl. Fig. S2. PA Y393 and S395 phospho-mutants do not affect PB1-PA interaction. (A) PB1, PB2 and the indicated PA Y393 and S395 phospho-mutants were expressed in 293T cells and 48 h.p.t. lysates were prepared. One aliquot of the lysate was used for the input controls, while the remaining extract was used for immunoprecipitation with anti-PA antibodies. The immunoprecipitated proteins were analyzed by immunoblotting as shown. The asterisk indicates a nonspecific band, and the right panel shows a schematic indicating the co-expressed proteins. Protein amounts of immunoprecipitated PA and PB1 were quantified using the ChemiDoc imaging system and normalized to tubulin. The PA/PB1 ratio of cells expressing the WT proteins was set to 1. Values from three independent experiments are shown and the medians are indicated, no statistically relevant changes were observed. (B) The experiment was performed as in (A) with the difference that also NP and a plasmid directing the expression of a vRNA was co-expressed.

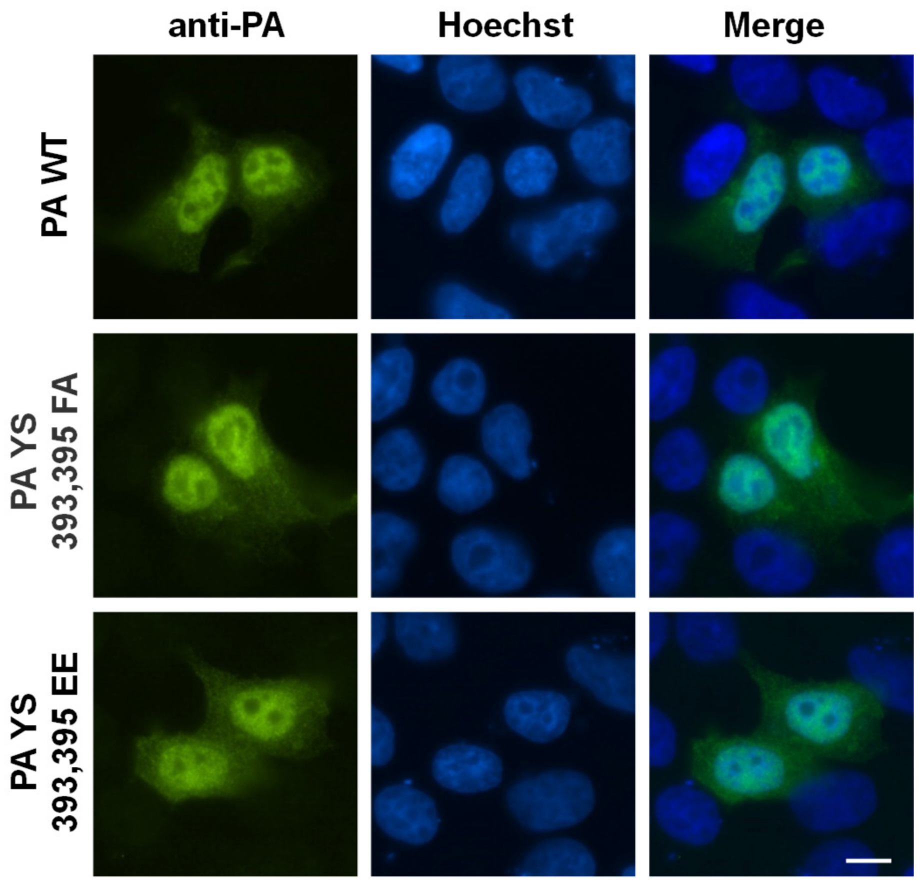

Suppl. Fig. S3. Phosphorylation of the PA subunit at Y393 and S395 does not alter its cellular localization. 293T cells were transfected with expression plasmids for PB1, PB2, PA WT and its mutants YS 393,395 FA or YS 393,395 EE as indicated. Cells were fixed and stained with anti-PA antibody (green). Nuclei were stained with Hoechst (blue). Pictures are representatives of three independent experiments, scale bar: 10 µm.

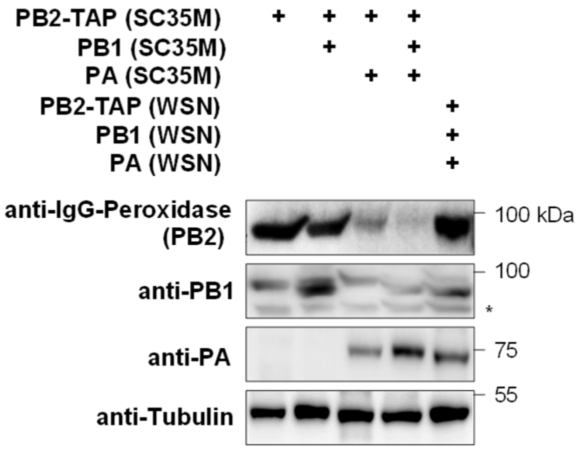

Suppl. Fig. S4. SC35M-PB2-TAP protein levels are reduced upon co-expression of the SC35M PA protein. 293T cells were transfected to express proteins encoded by SC35M or WSN as shown. After 2 days, lysates were prepared and Western blots were performed and viral proteins were detected with specific antibodies as shown. Since the TAP tag has two protein A binding sequences, PB2-TAP could be easily detected by incubation with a goat-anti-mouse antibody coupled to Peroxidase. The asterisk indicates a nonspecific band.
