# Supplementary material for: Phosphorylation of the PA subunit of influenza polymerase at Y393 prevents binding of the 5′-termini of RNA and polymerase function
Source: Sci Rep. 2023 Apr 29;13:7042. doi: 10.1038/s41598-023-34285-7 (PMC10148841; doi:10.1038/s41598-023-34285-7)
Supplement: Supplementary file 3 — Supplementary Table S2. [file 41598_2023_34285_MOESM3_ESM.docx]

**Phosphorylation of the PA subunit of influenza polymerase at Y393 prevents binding of the 5'-termini of RNA and polymerase function**

Lu Liu^1,2^, Ramakanth Madhugiri^2^, Vera Vivian Saul^1^, Susanne Bacher^1^, Michael Kracht^3^, Stephan Pleschka^2,4^ and M. Lienhard SCHMITZ^1,*^

^1^Institute of Biochemistry, Justus Liebig University Giessen (Germany), Member of the German Center for Lung Research (DZL)

^2^Institute of Medical Virology, Justus Liebig University Giessen (Germany)

^3^Rudolf-Buchheim-Institute of Pharmacology, Justus Liebig University, Giessen (Germany), Member of the German Center for Lung Research (DZL)

^4^German Center for Infection Research (DZIF), partner site Giessen (Germany)

**Supplementary Table S2.** Further information on reagents used in this study.

**Antibodies**

| **Primary antibody (clone)** | **Species** | **Supplier** | **Cat.No** |
| --- | --- | --- | --- |
| anti-IAV PA | rabbit pAb | Gene Tex | GTX118991 |
| anti-IAV PB1 | rabbit pAb | Gene Tex | GTX125923 |
| anti-IAV NP | rabbit pAb | Thermo Scientific | PA5-32242 |
| anti-β-Actin | rabbit pAb | Abcam | ab8227 |
| anti-α-Tubulin (12G10) | mouse mAb | DSHB | 12G10 |

| **Secondary antibody** | **Conjugated to** | **Supplier** |
| --- | --- | --- |
| goat-anti-mouse IgG | HRP | Dianova |
| goat-anti-rabbit IgG | HPR | Dianova |
| goat-anti-rabbit IgG | Alexa488 | Jackson ImmunoResearch |

**Further reagents**

| **Reagent** | **Supplier** | **Cat.No** |
| --- | --- | --- |
| λ phosphatase | New England Biolabs | P0753S |
| Phos-tag™ Acrylamide | FUJIFILM Wako | AAL-107M |
| Hoechst 33324 | Invitrogen | H1399 |
| Mowiol | Carl Roth | 81381 |

**DNA Oligonucleotides for site-directed mutagenesis**

| **Oligo name** | **Sequence (5´ to 3´)** |
| --- | --- |
| SC35M PB2-T471A-fw | CGACATGACCCCAAGTGCTGAGATGTCGCTGAGGGGA |
| SC35M PB2-T471A-rv | GCGACATCTCAGCACTTGGGGTCATGTCGGGTAATATCCCG |
| SC35M PB2-T471E-fw | CGACATGACCCCAAGTGAGGAGATGTCGCTGAGGGGA |
| SC35M PB2-T471E-rv | GCGACATCTCCTCACTTGGGGTCATGTCGGGTAATATCCCG |
| SC35M PA-YS393,395FA-fw | CAGCGATTTGAAACAGTTTGACGCCGATGAGCCAGAACAAAGATC |
| SC35M PA-YS393,395FA-rv | GTTCTGGCTCATCGGCGTCAAACTGTTTCAAATCGCTGACATCCTTG |
| SC35M PA-Y393E-fw | CAGCGATTTGAAACAGGAGGACAGCGATGAGCCAGAACAAAGATC |
| SC35M PA-Y393E-rv | GTTCTGGCTCATCGCTGTCCTCCTGTTTCAAATCGCTGACATCCTTG |
| SC35M PA-S395E-fw | CAGCGATTTGAAACAGTATGACGAGGATGAGCCAGAACAAAGATC |
| SC35M PA-S395E-rv | GTTCTGGCTCATCCTCGTCATACTGTTTCAAATCGCTGACATCCTTG |
| SC35M PA-YS393,395EE-fw | CAGCGATTTGAAACAGGAGGACGAGGATGAGCCAGAACAAAGATC |
| SC35M PA-YS393,395EE-rv | GTTCTGGCTCATCCTCGTCCTCCTGTTTCAAATCGCTGACATCCTTG |
| WSN-PA-YS393,395FA-fw | AGGCGATTTGAAGCAATTTGATGCTGATGAACCAGAATTGAGGTC |
| WSN-PA-YS393,395FA-rv | ATTCTGGTTCATCAGCATCAAATTGCTTCAAATCGCCTACATCTTTA |
| WSN-PA-Y393E-fw | AGGCGATTTGAAGCAAGAGGATAGTGATGAACCAGAATTGAGGTC |
| WSN-PA-Y393E-rv | ATTCTGGTTCATCACTATCCTCTTGCTTCAAATCGCCTACATCTTTA |
| WSN-PA-S395E-fw | AGGCGATTTGAAGCAATATGATGAGGATGAACCAGAATTGAGGTC |
| WSN-PA-S395E-rv | ATTCTGGTTCATCCTCATCATATTGCTTCAAATCGCCTACATCTTTA |
| WSN-PA-YS393,395EE-fw | AGGCGATTTGAAGCAAGAGGATGAGGATGAACCAGAATTGAGGTC |
| WSN-PA-YS393,395EE-rv | ATTCTGGTTCATCCTCATCCTCTTGCTTCAAATCGCCTACATCTTTA |

**Primers for primer extension assay**

| **Oligo name** | **Sequence (5´ to 3´)** |
| --- | --- |
| CAT vRNA | CGCAAGGCGACAAGGTGCTGA |
| CAT mRNA and cRNA | ATGTTCTTTACGATGCGATTGGG |
| 5S rRNA | TCCCAGGCGGTCTCCCATCC |
