# Supplementary material for: Phosphorylation of the PA subunit of influenza polymerase at Y393 prevents binding of the 5′-termini of RNA and polymerase function
Source: Sci Rep. 2023 Apr 29;13:7042. doi: 10.1038/s41598-023-34285-7 (PMC10148841; doi:10.1038/s41598-023-34285-7)
Supplement: Supplementary file 4 — Supplementary Information 4. [file 41598_2023_34285_MOESM4_ESM.pdf]

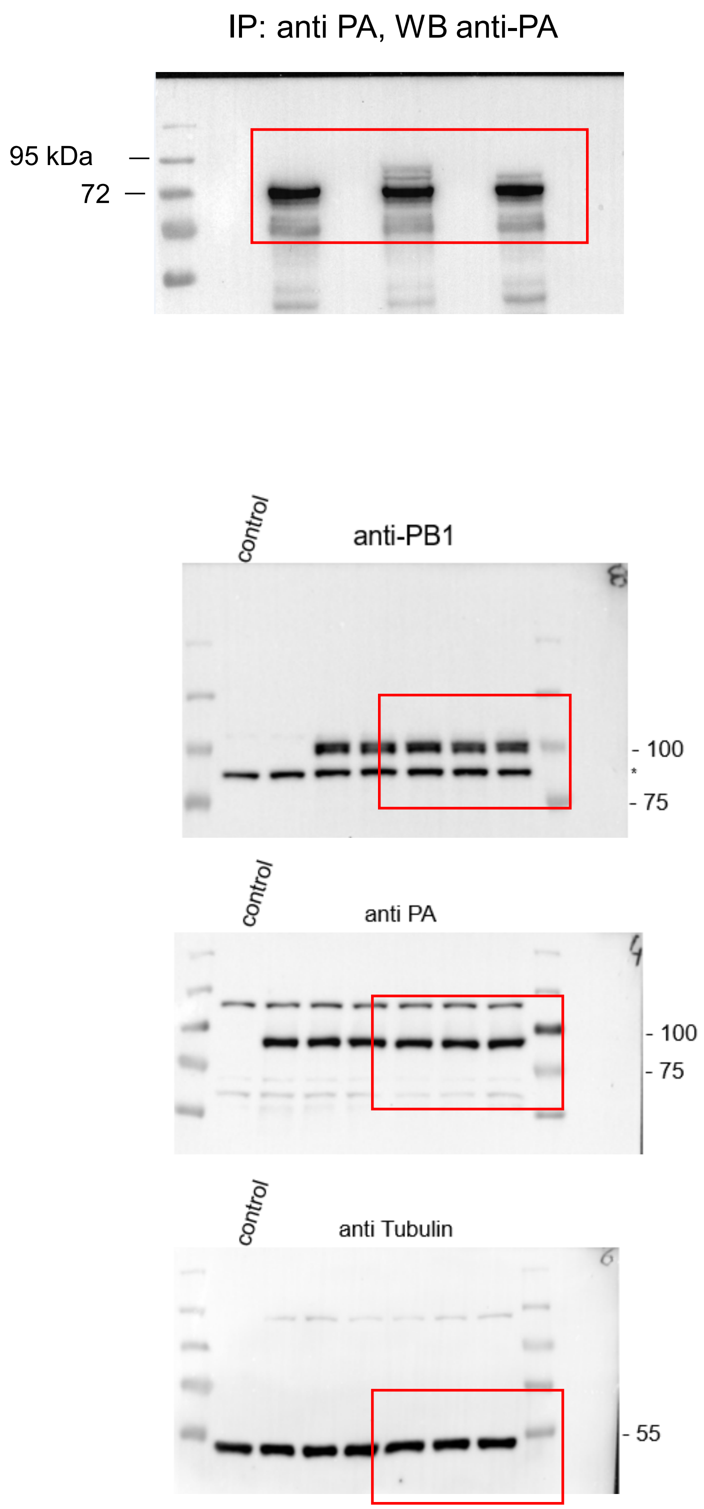
IP: anti PA, WB anti-PA
95 kDa
72
control
anti-PB1
- 100
*
- 75
control
anti PA
- 100
- 75
control
anti Tubulin
- 55

Fig. 2A

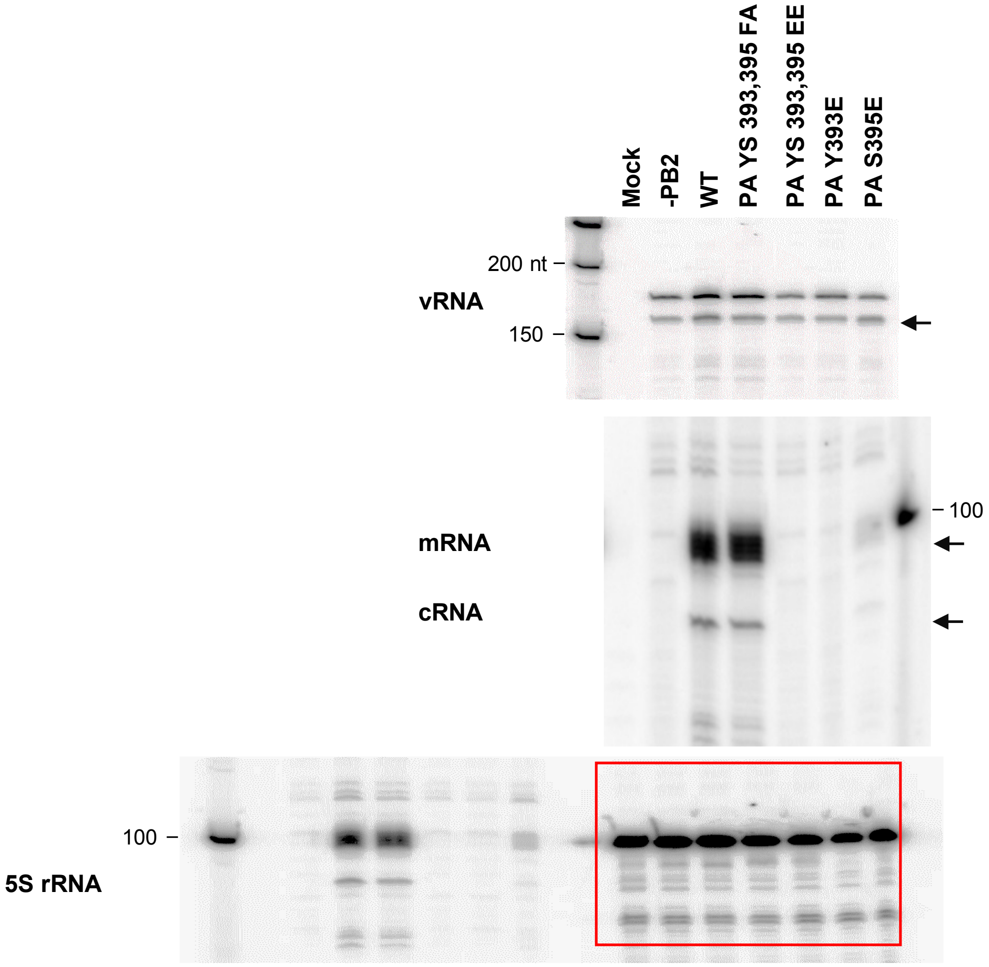

Mock
-PB2
WT
PA YS 393,395 FA
PA YS 393,395 EE
PA Y393E
PA S395E
200 nt
150
vRNA
100
mRNA
cRNA
100
5S rRNA

Fig. 3C

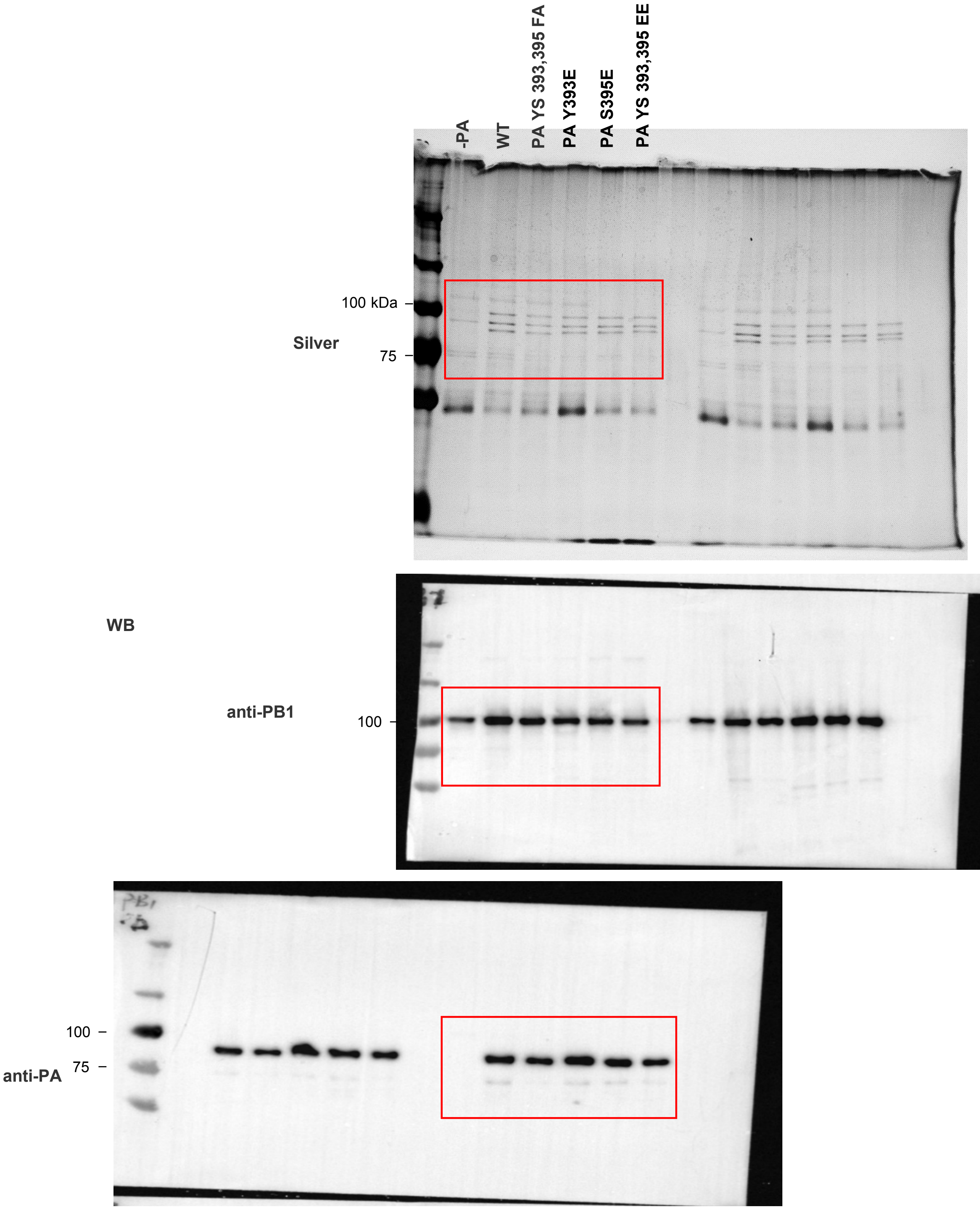
-PA
WT
PA YS 393,395 FA
PA Y393E
PA S395E
PA YS 393,395 EE
100 kDa
Silver
75
WB
anti-PB1
100
100
anti-PA
75

Fig. 4B

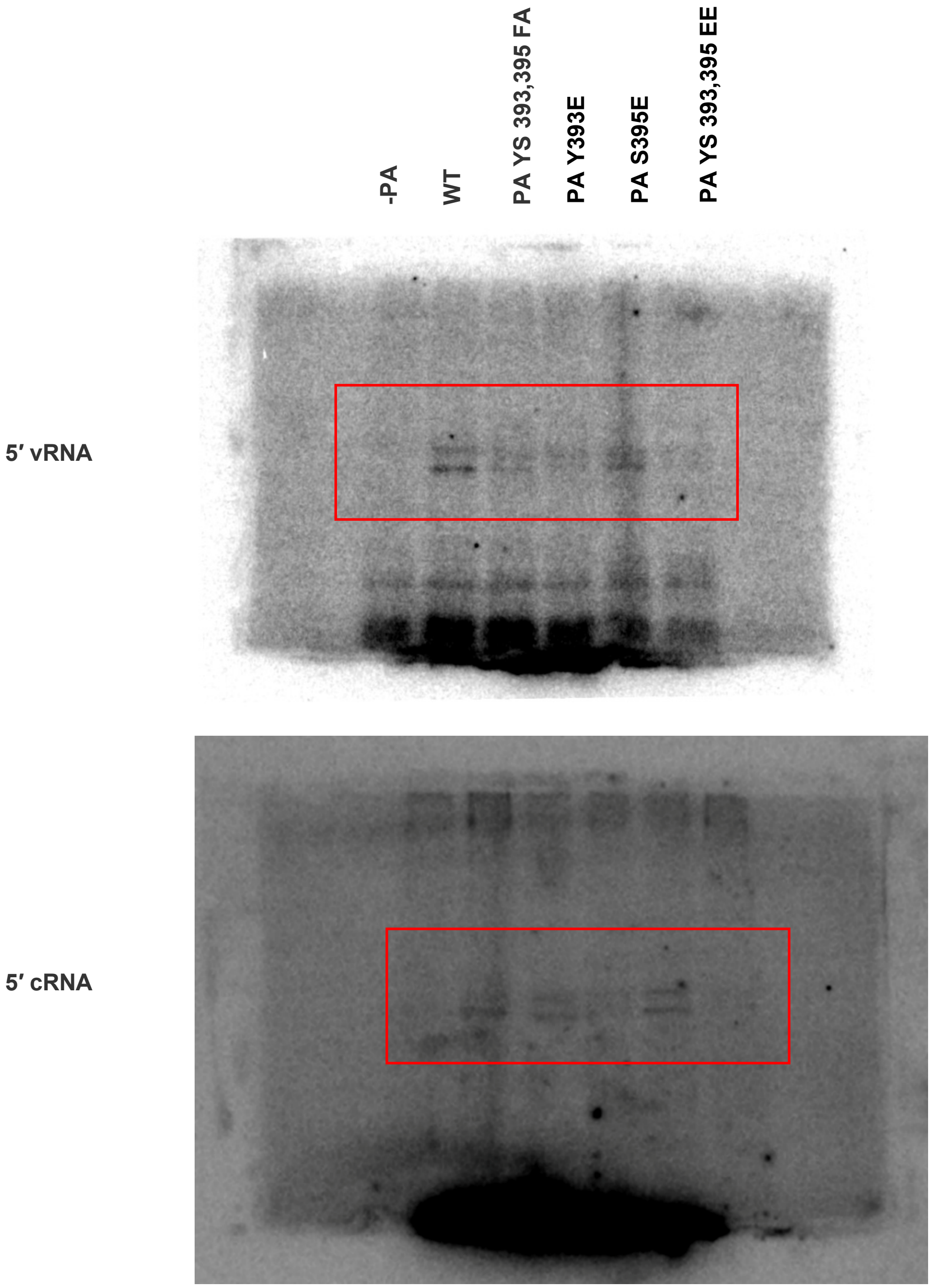
-PA
WT
PA YS 393,395 FA
PA Y393E
PA S395E
PA YS 393,395 EE
5′ vRNA
5′ cRNA

Fig. 4C

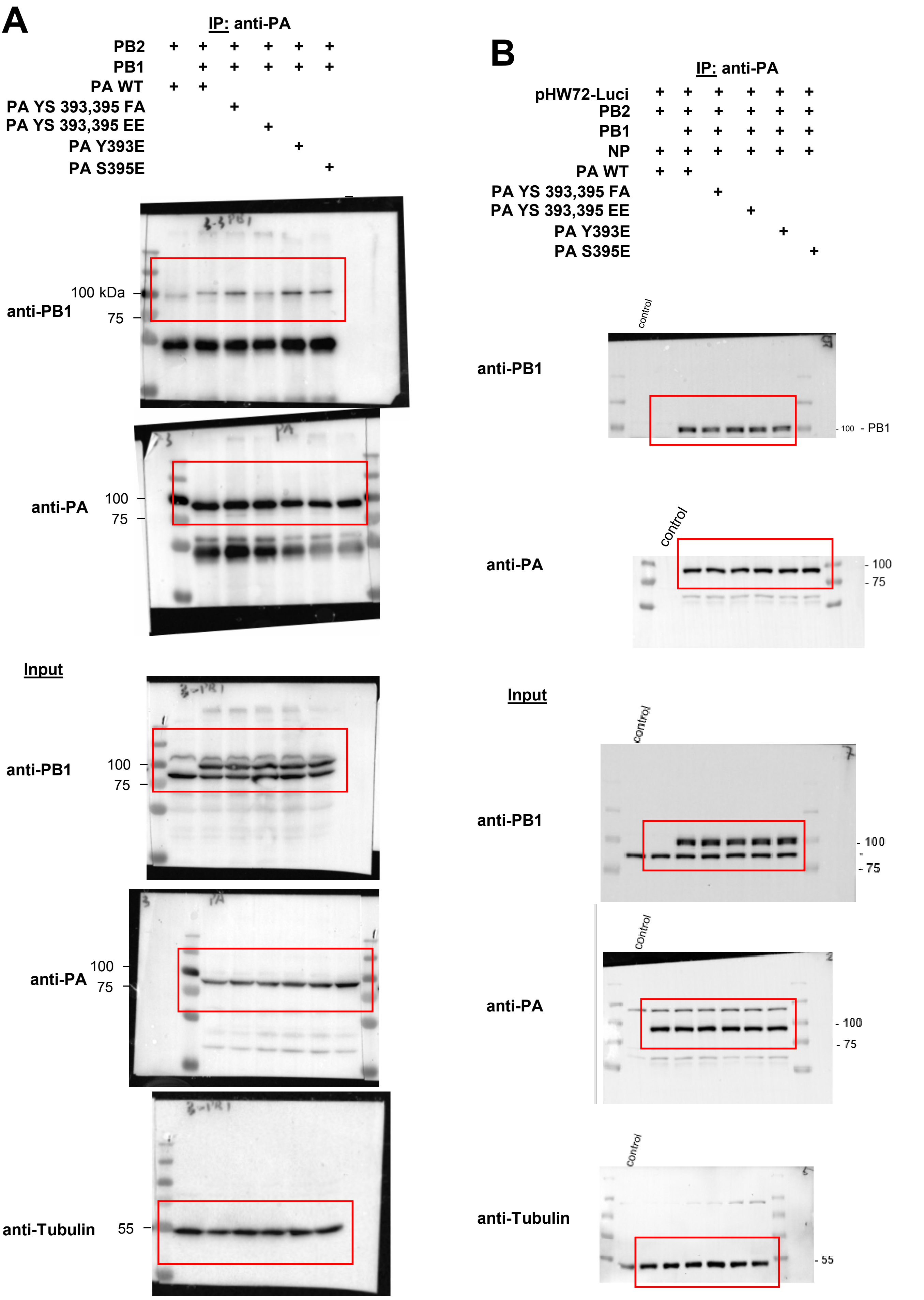
A
IP: anti-PA
PB2
PB1
PA WT
PA YS 393,395 FA
PA YS 393,395 EE
PA Y393E
PA S395E
anti-PB1
100 kDa
75
anti-PA
100
75
Input
anti-PB1
100
75
anti-PA
100
75
anti-Tubulin
55
B
IP: anti-PA
pHW72-Luci
PB2
PB1
NP
PA WT
PA YS 393,395 FA
PA YS 393,395 EE
PA Y393E
PA S395E
control
anti-PB1
100
PB1
control
anti-PA
100
75
Input
control
anti-PB1
100
75
control
anti-PA
100
75
control
anti-Tubulin
55

Fig. S2

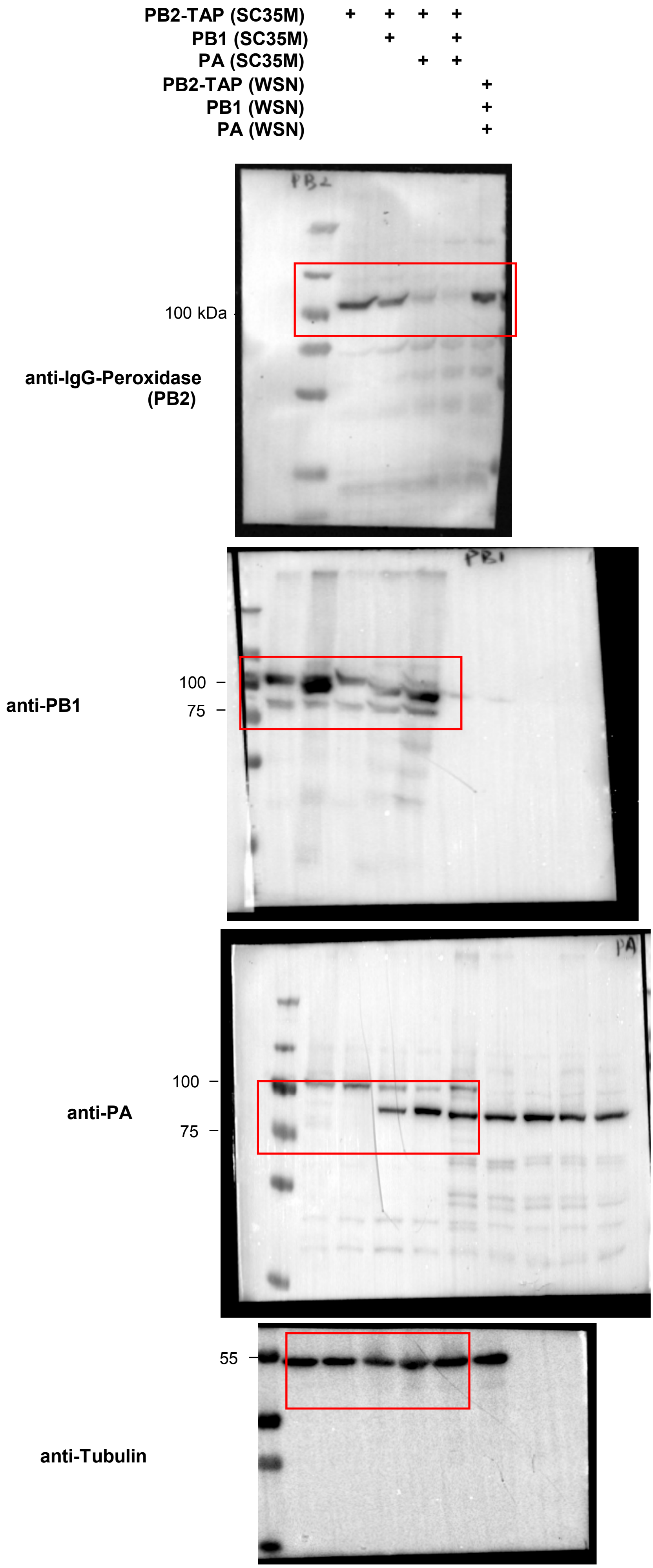
PB2-TAP (SC35M) + + + +
PB1 (SC35M) + +
PA (SC35M) + +
PB2-TAP (WSN) +
PB1 (WSN) +
PA (WSN) +
100 kDa
anti-IgG-Peroxidase (PB2)
100
75
anti-PB1
100
75
anti-PA
55
anti-Tubulin

Fig. S4
